# TNFα induced up-regulation of Na^+^,K^+^,2Cl^−^ cotransporter NKCC1 in hepatic ammonia clearance and cerebral ammonia toxicity

**DOI:** 10.1038/s41598-017-07640-8

**Published:** 2017-08-11

**Authors:** Vitaly I. Pozdeev, Elisabeth Lang, Boris Görg, Hans J. Bidmon, Prashant V. Shinde, Gerald Kircheis, Diran Herebian, Klaus Pfeffer, Florian Lang, Dieter Häussinger, Karl S. Lang, Philipp A. Lang

**Affiliations:** 1 Department of Gastroenterology, Hepatology, and Infectious Diseases, Heinrich-Heine-University Düsseldorf, Universitätsstr. 1, 40225 Düsseldorf, Germany; 2 Department of Molecular Medicine II, Medical Faculty, Heinrich Heine University, Universitätsstr. 1, 40225 Düsseldorf, Germany; 3 C.&O. Vogt Institute for Brain Research, Heinrich-Heine-University Düsseldorf, 40225 Düsseldorf, Germany; 4 Department of General Pediatrics, Neonatology, and Pediatric Cardiology, Heinrich Heine University Düsseldorf, 40225 Düsseldorf, Germany; 5 Institute of Medical Microbiology and Hospital Hygiene, Heinrich-Heine- University Düsseldorf, 40225 Duesseldorf, Germany; 6 Department of Internal Medicine III, Eberhard-Karls Universitaet Tuebingen, Tuebingen, Germany; 7 Institute of Immunology, Medical Faculty, University of Duisburg-Essen, Hufelandstr. 55, Essen, 45147 Germany

## Abstract

The devastating consequences of hepatic failure include hepatic encephalopathy, a severe, life threatening impairment of neuronal function. Hepatic encephalopathy is caused by impaired hepatic clearance of NH_4_^+^. Cellular NH_4_^+^ uptake is accomplished mainly by the Na^+^,K^+^,2Cl^−^ cotransporter. Here we show that hepatic clearance of NH_4_^+^ is impaired in TNFα deficient as well as TNFR1&TNFR2 double knockout mice, which both develop hyperammonemia. Despite impaired hepatic clearance of NH_4_^+^, TNFα deficient mice and TNFR1 deficient mice were protected against acute ammonia intoxication. While 54% of the wild-type mice and 60% of TNFR2 deficient mice survived an NH_4_^+^ load, virtually all TNFα deficient mice and TNFR1 deficient mice survived the treatment. Conversely, TNFα treatment of wild type mice sensitized the animals to the toxic effects of an NH_4_^+^ load. The protection of TNFα-deficient mice against an NH_4_^+^ load was paralleled by decreased cerebral expression of NKCC1. According to the present observations, inhibition of TNFα formation and/or NKCC1 may be strategies to favorably influence the clinical course of hepatic encephalopathy.

## Introduction

Ammonia detoxification in the liver is critical to prevent toxic effects in the brain^1^. Consequently, increased ammonia levels in the peripheral blood are associated with hepatic encephalopathy, a devastating clinical condition following liver failure^1^. Ammonia is metabolized in the liver to urea and glutamine^2^. The rate limiting enzyme for hepatic urea synthesis is carbamoylphosphate synthetase (Cps-1), which only exhibits a low affinity for ammonia^3^. Hence, high ammonia concentrations are provided by glutaminase activity to feed ammonia into the urea cycle^2, 3^. Remaining ammonia, which escapes the urea cycle, is taken up by perivenous scavenger cells and metabolized to glutamine by glutamine synthetase^4–6^. This high affinity ammonia metabolizing mechanism prevents toxic increase of ammonia in the circulation. Consequently, defects in these perivenous scavenger cells result in increased ammonia concentrations in the blood^4, 7^. Furthermore, defects in hepatic glutamine synthetase activity trigger hyperammonemia and behavioral changes^8, 9^.

Hepatic encephalopathy (HE) is the clinical manifestation of a low grade cerebral edema associated with oxidative/nitrosative stress in brain tissue^10^. The severity of hepatic encephalopathy correlates with increased levels of ammonia in peripheral blood^11–13^. Ammonia induces senescence in astrocytes which may explain persistence of cognitive impairment after resolution of an acute HE attack^14^. Ammonia compromises astrocyte-dependent potassium buffering, thereby increasing extracellular potassium concentration and enhancing Na^+^,K^+^,2Cl^−^ cotransporter (NKCC1) activity^15^.

NKCC1 accomplishes cellular NH_4_^+^ uptake^16^. Accordingly, NKCC1 deficient mice showed reduced susceptibility towards ammonia intoxication^15^. NKCC1 expression is upregulated by tumor necrosis factor alpha (TNFα)^17^. TNFα and interleukin 1 beta (Il-1b) have been shown to be upregulated in brain tissue during acute liver failure in mice^18^. Ammonia and TNFα levels are both increased in circulating blood of patients with hepatic encephalopathy^11–13^. Previous studies suggested that TNFα may increase ammonia levels in patients^13^. However, during grade 1 and 2 hepatic encephalopathy no to little increase of ammonia concentrations in the blood stream is observed^11^.

In the liver TNFα may induce apoptosis via tumor necrosis factor receptor 1 (TNFR1) and consequently induce liver damage during septic shock and infections^19–21^. TNF mediated liver damage may trigger hyperammonemia and thus further aggravate hepatic encephalopathy. Consistently, TNF blockade by etanercept in an acute liver failure model reduced hepatic damage and hyperammonemia^22^. While a role for TNFα during liver damage is recognized, its role on cognitive functions such as memory formation mediated through astrocytes has only recently been appreciated^23^. A role of TNFα during ammonia toxicity has been suggested, but a detailed understanding of its effects during hyperammonemia remained elusive.

Here we provide evidence suggesting that basal expression of TNFα upregulates Cps-1, a key enzyme in hepatic NH_4_^+^ metabolism. Also, TNFα deficient mice exhibited elevated ammonia levels when compared to control mice. In the brain TNFα triggered NKCC1 expression and augmented NH_4_^+^ toxicity. Along those lines TNFR1-deficient animals were resistant to acute ammonia intoxication. Hence, therapeutic regimens targeting TNFα, TNFR1 or NKCC1 may counteract hepatic encephalopathy.

## Results

### Ammonia levels in the blood were enhanced and expression of hepatic Cps-1 decreased in TNFα deficient animals

**Figure 1:**
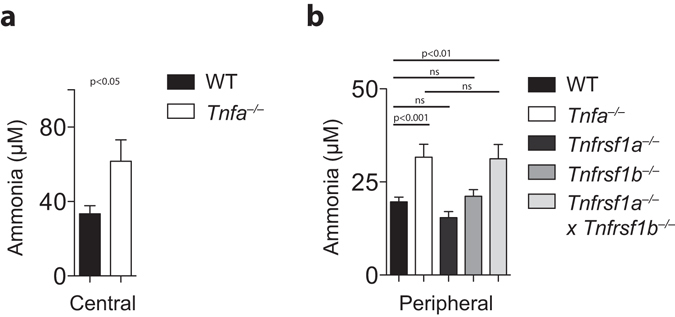
Hyperammonemia in TNFα- and TNFR1/TNFR2-deficient animals. **(a)** Ammonia levels were measured in blood samples harvested by cardiac puncture, central blood (right panel, n = 12) of WT and *Tnfa* ^−/−^ mice. (**b)** Ammonia levels were measured in blood samples harvested from the retro orbital vein sinus of WT (n = 33), *Tnfa* ^−/−^ (n = 16), *Tnfrsf1a* ^−/−^ (n = 17), *Tnfrsf1b* ^−/−^ (n = 18), and *Tnfrsf1a* ^−/−^ *Tnfrsf1b* ^−/−^ animals (n = 9).

**Figure 2:**
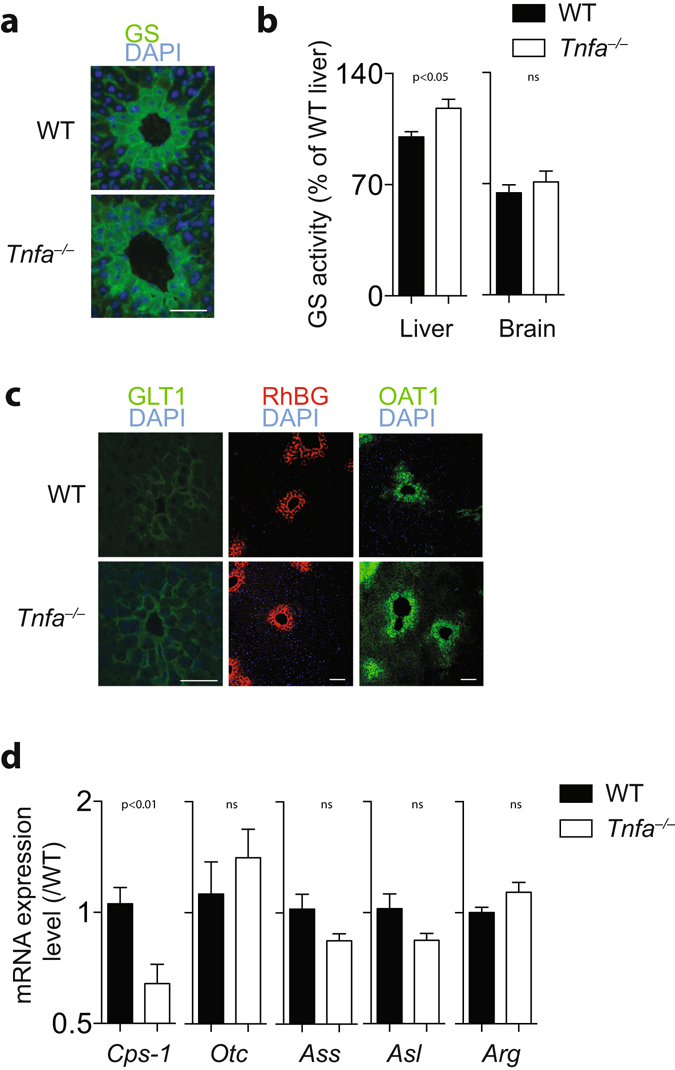
Intact liver structure but reduced hepatic expression of CPS-1 in TNFα deficient animals. **(a)** Sections from snap frozen liver tissue harvested from WT and *Tnfa* ^−/−^ mice were stained with anti-GS antibodies (One representative of n = 6 is shown, Scale bar = 25 µm). **(b)** GS activity was measured in liver tissue (left panel, n = 6), and whole brain tissue (right panel, n = 3) from WT and *Tnfa* ^−/−^ mice. **(c)** Sections from snap frozen liver tissue from WT and *Tnfa* ^−/−^ mice were stained with anti-GLT1 (left panels, scale bar = 25 µm), anti-RhBG (middle panels, scale bar = 100 µm), and anti-OAT1 antibodies (right panels, scale bar = 100 µm). One representative out of n = 6 is shown. **(d)** RNA expression levels of *Cps-1* (n = 9), *Otc* (n = 6), *Ass* (n = 6), *Asl* (n = 6), and *Arg* (n = 6) were determined in liver tissue from WT and *Tnfa* ^−/−^ mice.

Surprisingly, blood concentrations of ammonia were increased in TNFα deficient animals (Fig. 1a). This increased ammonia concentration was dependent on both, TNFR1 and tumor necrosis factor receptor 2 (TNFR2) signaling, as only TNFR1&TNFR2 double knockout showed also increased ammonia levels in the blood stream (Fig. 1b). Next, we investigated whether TNFα affects ammonia metabolism in the liver. Absence of hepatic glutamine synthetase results in hyperammonemia and increased behavioral activity^9^. However, localization of hepatic glutamine synthetase was not different between TNFα-deficient animals and WT mice (Fig. 2a). Hepatic glutamine synthetase activity in *Tnfa* ^−/−^ animals was even significantly increased when compared to WT controls (Fig. 2b). Moreover, the expressions of ornithine aminotransferase, Rhesus family B glycoprotein, and excitatory amino acid transporter 2 (EAAT2/GLT-1) were similar in WT- and TNFα-deficient liver tissue (Fig. 2c). These data suggest that expression of ammonia detoxifying enzymes in perivenous scavenger cells is not affected by absence of TNFα. However, expression of the rate limiting enzyme for the urea cycle, Cps-1 was significantly reduced in *Tnfa* ^−/−^ liver tissue, while expression levels of other urea cycle enzymes were not different (Fig. 2d). These data may indicate that reduced *Cps-1* gene expression contributed to hyperammonemia in the absence of TNFα.

### Cerebral aquaporin 4, 8 and 9 transcript levels, tyrosine-nitrated proteins and oxidized RNA are similar in wild-type and TNFα deficient animals

**Figure 3:**
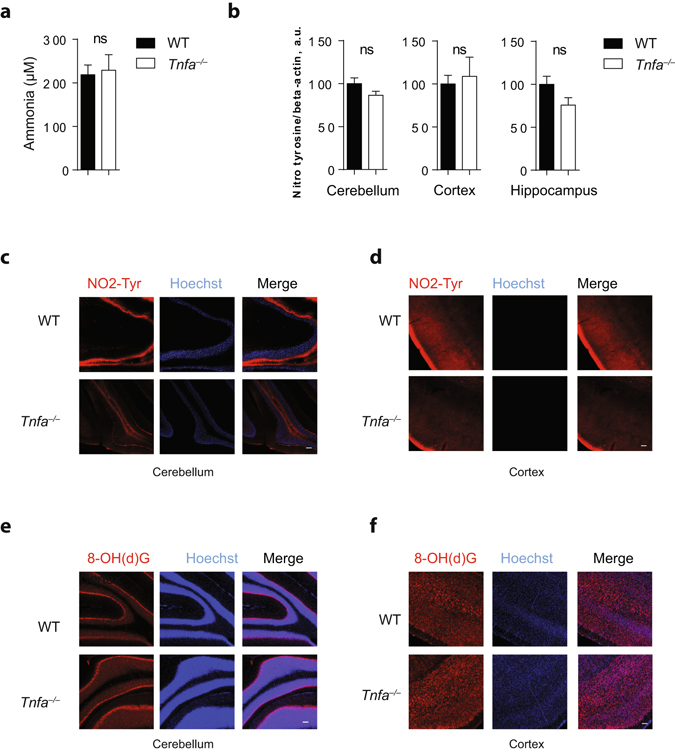
Normal tyrosine nitration and RNA oxidation in brain tissues from *Tnfa* ^*−/−*^ mice. **(a)** Ammonia levels were measured in cerebrospinal fluid samples of WT and *Tnfa* ^−/−^ mice (n = 4–6). **(b)** Protein lysates harvested from the cerebellum of WT and *Tnfa* ^*−/−*^ mice were blotted and stained using anti-nitrotyrosine and anti-beta-actin antibodies, panel illustrates the densitometric analysis of nitrotyrosine/beta-actin (n = 17). **(c**–**d)** Sections from snap frozen (**c**) cerebellum or (**d**) cortex of WT and TNFα deficient mice were stained with anti-nitrotyrosine antibodies (red) and Hoechst (blue). One representative of n = 6 is shown, scale bar = 100 µm. **(e**–**f)** Sections from snap frozen (**e**) cerebellum or (**f**) cortex of WT and TNFα deficient mice were stained with anti-8-OH(d)G antibodies (red) and Hoechst (blue). One representative of n = 6 is shown, scale bar = 100 µm.

Ammonia transport can be accomplished by aquaporins^24, 25^. Furthermore, aquaporin-4 can be induced by ammonia and trigger cerebral edema^26, 27^. We did, however, not find differences of aquaporin expression in the cerebellum or the cortex between WT and TNFα deficient animals (Supplementary Fig. 1a,b). Moreover, ammonia levels in the cerebrospinal fluid were similar in WT and TNFα knockout animals and comparable to concentrations found previously (Fig. 3a)^28^. Ammonia can cause protein tyrosine nitration and RNA oxidation in the brain^9, 10^. No difference in protein tyrosine nitration was observed by Western blot analysis between WT and TNFα knockout animals (Fig. 3b). Histological analysis of brain tissue from WT and *Tnfa* ^−/−^ animals revealed similar nitrotyrosine abundance (Fig. 3c–d). The levels of oxidized RNA were again similar in WT and TNFα deficient animals (Fig. 3e–f).

### TNFα-deficient mice are protected against acute ammonia intoxication

**Figure 4:**
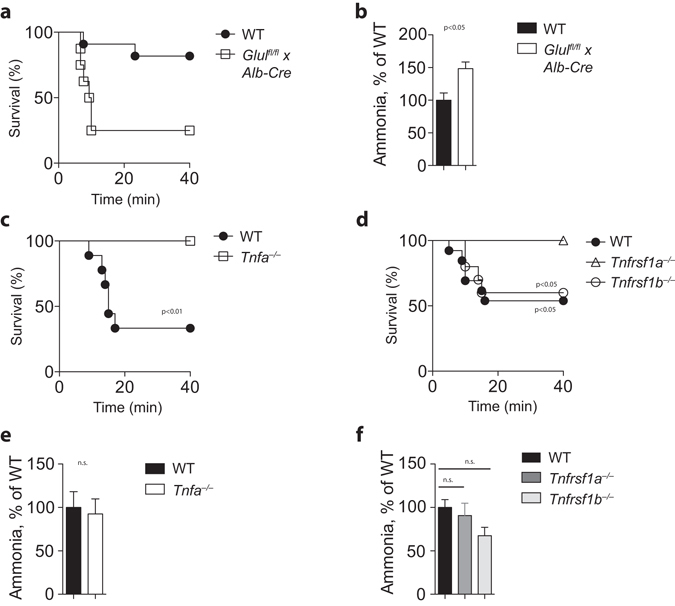
*Tnfa* ^−/−^ mice are protected against acute ammonia intoxication. **(a)** WT and *Glul* ^*fl/fl*^ × *Alb-Cre* ^+^ mice were challenged with 12 mmol/kg ammonium acetate in PBS following monitoring of survival (n = 8–11). **(b)** WT and *Glul* ^*fl/fl*^ × *Alb-Cre* ^+^ mice were challenged with 8 mmol/kg ammonium acetate in PBS, ammonia levels were measured in blood samples harvested from the retro-orbital venous sinus (n = 5–6). **(c)** Survival was monitored in WT and *Tnfa* ^−/−^ mice after challenge with 14 mmol/kg ammonium acetate in PBS (n = 8–9). (**d)** WT, *Tnfrsf1a* ^−/−^, and *Tnfrsf1b* ^−/−^ mice were challenged with 14 mmol/kg ammonium acetate in PBS following monitoring of their survival (n = 10–13). **(e)** WT and *Tnfa* ^−/−^ mice were challenged with 8 mmol/kg ammonium acetate in PBS and ammonia levels were measured in blood samples harvested from the retro-orbital venous sinus (n = 9). (**f)** WT, *Tnfrsf1a* ^−/−^, and *Tnfrsf1b* ^−/−^ mice were challenged with 8 mmol/kg ammonium acetate in PBS and ammonia levels were measured in blood samples harvested from the retro-orbital venous sinus (n = 7–9).

**Figure 5:**
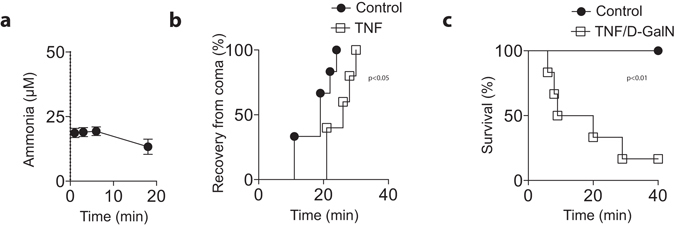
TNFα increases susceptibility towards ammonia toxicity. **(a)** Ammonia concentrations were measured from retro-orbital venous sinus after i.v. injection of 200 ng TNF (n = 3). **(b)** C57Bl/6 animals were challenged intravenously with either 200 ng TNF or vehicle. After 3 hours 12 mmol/kg ammonium acetate in PBS was injected intraperitoneally followed by measurement of the coma time (n = 5–6). **(c)** C57Bl/6 mice were injected with D-Gal and after 15 minutes with 200 ng TNF. After 3 hours animals were challenged with 12 mmol/kg ammonium acetate in PBS along with a control group (n = 6).

Impaired hepatic glutamine synthesis results in hyperammonemia similar to TNFα deficient animals^9^. Consistently, when *Glul* ^*fl/fl*^ *x Alb-Cre* ^+^ animals were challenged with a sublethal dose of ammonia, animals were more susceptible towards ammonia toxicity when compared to WT controls (Fig. 4a). We observed impaired ammonia clearance after ammonia acetate challenge in *Glul* ^*fl/fl*^ *x Alb-Cre* ^+^ mice (Fig. 4b). In sharp contrast, when we challenged TNFα-deficient mice with a dose of ammonia lethal to WT mice, we observed that TNFα-deficient mice were resistant against acute ammonia intoxication (Fig. 4c). We next wondered whether TNFα triggers its toxic effect in the brain specifically through one TNFR. When we challenged WT, *Tnfrsf1a* ^*−/−*^, and *Tnfrsf1b* ^*−/−*^ animals with ammonia, reduced susceptibility was only seen in TNFR1 animals, while WT and TNFR2 animals exhibited similar effects following acute ammonia intoxication (Fig. 4d). We observed no significant difference in ammonia concentration after ammonia acetate challenge between WT and TNF deficient mice or between WT, TNFR1 knockout, and TNFR2 knockout animals (Fig. 4e,f). To further investigate the role of TNFα during ammonia toxicity, we challenged animals with TNFα. In absence of D-Gal, TNFα did not trigger liver damage and did not affect ammonia concentrations (Fig. 5a). However, the coma time following acute ammonia intoxication was significantly increased after challenge with TNFα (Fig. 5b). Notably, this was a transient effect as 24 h after TNF injection we did not observe increased coma time (Supplementary Fig. 2). Furthermore, when we challenged animals with TNFα in addition with D-Gal followed by challenge with ammonia acetate, we observed high susceptibility of animals towards ammonia toxicity (Fig. 5c). Taken together, these data indicate that TNFα promotes ammonia toxicity.

### TNFα up-regulates NKCC1 expression in brain tissues

**Figure 6:**
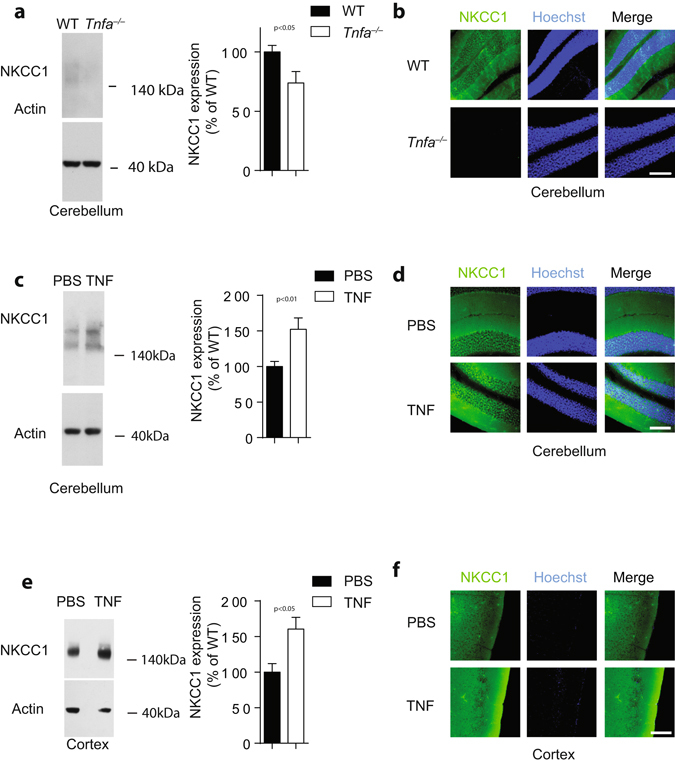
TNFα regulates expression of NKCC1 in brain tissue. **(a)** Protein lysates harvested from the cerebellum of WT and *Tnfa* ^−/−^ mice were blotted and stained using anti-NKCC1 (upper panel) and anti-beta-actin (lower panel) antibodies (One representative of n = 12 is shown cropped). Right panel illustrates the densitometry of NKCC1/beta-actin (n = 12). Full-size images are presented in Supplementary Fig. 3a. (**b**) Sections from snap frozen cerebellum of WT and TNFα deficient mice were stained with anti-NKCC1 antibodies (green) and Hoechst (blue). One representative of n = 6 is shown, scale bar = 100 µm. **(c**–**f)** C57Bl/6 mice were treated with 200ng TNF. After 3 h, animals were killed and **(c**, **d)** cerebellum and **(e**, **f)** cortex was harvested. **(c)** Protein lysates from cerebellum were prepared, blotted and stained with anti-NKCC1 and anti-beta-actin antibodies (left panels, one representative of n = 8 is shown cropped). Right panels illustrate the mean ± S.E.M. of the densitometric analysis of NKCC1/beta-actin (n = 8). Full-size images are presented in Supplementary Fig. 3b. (**d)** Sections from snap frozen cerebellum were stained with anti-NKCC1 antibodies (green) and Hoechst (blue, n = 6). **(e)** Protein lysates from cortex were prepared, blotted and stained with anti-NKCC1 and anti-beta-actin antibodies (left panels, one representative of n = 8 is shown cropped). Right panels illustrate the mean ± S.E.M. of the densitometric analysis of NKCC1/beta-actin (n = 8). Full-size images are presented in Supplementary Fig. 3c. (**f**) After 3 h, sections from snap frozen cortex were stained with anti-NKCC1 antibodies (green) and Hoechst (blue, n = 6).

**Figure 7:**
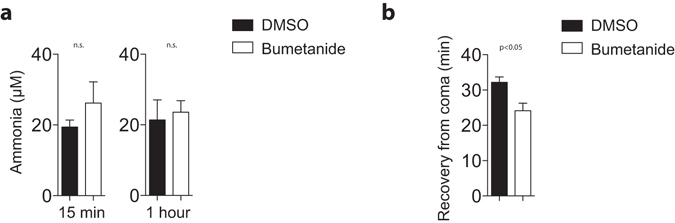
Inhibition of NKCC1 alleviates acute ammonia toxicity. **(a)** Ammonia concentrations were measured from retro-orbital venous sinus after i.p. injection of 30 mg/kg bumetanide (n = 5). **(b)** C57Bl/6 animals were treated intraperitoneally with either 30 mg/kg Bumetanide or vehicle. After 5 minutes 14 mmol/kg ammonium acetate in PBS was injected intraperitoneally followed by measurement of the duration of intoxication (time until recovery from coma) (n = 5–7).

Ammonia toxicity can be influenced by NKCC1 expression in astrocytes^30, 31^, which accomplishes cellular NH_4_^+^ uptake^16, 29, 30^ and buffers extracellular potassium^15, 32, 33^. Hence, we wondered whether TNFα might trigger NKCC1 expression in astrocytes. According to Western blotting, cerebellar NKCC1 expression was reduced in TNFα-deficient animals (Fig. 6a). Moreover, histological analyses of NKCC1 expression in brain tissue uncovered its expression, which was reduced in the absence of TNFα (Fig. 6b). Conversely, injection of TNFα significantly increased NKCC1 expression in cerebellum (Fig. 6c,d) and cerebral cortex (Fig. 6e,f). We were wondering if pharmacological blockade of NKCC1 could alter ammonia levels or could modulate toxic effects of ammonia acetate *in vivo*. When we administrated intraperitoneally bumetanide (30 mg/kg), we did not observe significant differences in blood ammonia levels between bumetanide or vehicle treated WT mice (Fig. 7a). However, when bumetanide and vehicle treated WT mice were challenged with 14 mmol/kg of ammonia acetate, coma duration in bumetanide treated mice was significantly shorter compared to vehicle treated mice (Fig. 7b). Taken together these data indicate that TNFα up-regulates cerebral expression of NKCC1, which in turn significantly contributes to the pathophysiology of acute ammonia intoxication.

## Discussion

In this study we disclosed a critical role of TNFα for ammonia metabolism and toxicity. TNFα is important for metabolizing ammonia in the liver. Consequently, TNFα-deficient animals exhibited hyperammonemia. Moreover, TNF triggers NKCC1 expression in brain tissue and this may promote toxic effects of ammonia in the brain. Hence, TNFα-deficient and TNFR1-deficient animals were protected against acute ammonia intoxication.

Ammonia metabolism in the liver depends on two major mechanisms, the urea cycle on the one hand and glutamine production in the perivenous scavenger cells on the other hand. During liver damage with lipopolysaccharide (LPS), nitration of glutamine synthetase decreases enzyme activity and thus leads to hyperammonemia^8, 31^. LPS mediated liver damage and toxicity depends largely on TNFα^19, 20^, suggesting that TNFα may be detrimental for productive ammonia metabolism. However, our data indicate, that basal TNFα signaling supports ammonia detoxification in liver tissue. Physiological levels of TNFα may promote *Cps-1* expression and consequently ammonia metabolism in the liver. However, excess TNFα production would cause liver damage, limit hepatic ammonia metabolism, to foster development of hepatic encephalopathy^8, 13, 19, 20, 31^. Indeed in a model of acute liver failure, application of etanercept could alleviate liver damage, hyperammonemia, and toxic effects in the brain^18, 22^. However, previous studies did not find increased ammonia concentrations in patients with grade 1 and grade 2 hepatic encephalopathy^11^. However, increased TNFα concentrations were observed in grade 1 and 2 HE patients^12, 13^. Moreover, high volume plasma exchange in patients decreases production of pro-inflammatory cytokines by monocytes and improves survival of patients with acute liver failure^32, 33^. These data suggest that TNF might modulate ammonia toxicity. Our data indicate that TNFα mediates NKCC1 expression and that NKCC1 increases the susceptibility to ammonia toxicity. Moreover TNFα can cause cerebral excitotoxicity by activation of glutaminase and stimulation of glutamate release^34^. Extracellular glutamate accumulates thus inducing excitotoxicity^35^. Toxic effects of ammonia in the brain are mediated by NMDA receptors and pharmacological inhibition of those receptors fosters survival during acute ammonia intoxication^36–40^. TNFα enhances toxic effects of glutamate and induces cell death^41^. Rats with chronic hyperammonemia are protected against acute ammonia toxicity due to reduced activity of the NMDA-NO-cGMP pathway^42–44^.

It is tempting to speculate that blockade of TNFR1 would be a superior therapeutic approach in hepatic encephalopathy, because blockade of TNFR1 would reduce toxic effects in brain tissue without affecting ammonia metabolism in the liver.

TNFα, IL-1β, interleukin 6 (IL-6) are produced in brain in animal models of chronic hyperammonemia^45–48^. Also, during acute liver intoxication or liver ischemia IL-1β, IL-6, and TNFα levels are enhanced in blood^49^. Moreover, microglia activation is observed in animal models of acute and chronic hepatic encephalopathy^22, 45–47, 50, 51^. In post mortem brain tissue from patients with liver cirrhosis and hepatic encephalopathy genes associated with microglia activation but not pro-inflammatory cytokines were up-regulated^52^. Pro-inflammatory cytokines may be produced by circulating lymphocytes such as neutrophils, which may produce TNFα in dependence of TLR4, TLR9 and ammonia^53, 54^.

Recent evidence indicated that encephalitic TNFα can result in memory deficits due to signaling through astrocytes^23^. Pharmacological blocking of TNFα by Infliximab improves learning ability and coordination in rats with HE^45^. Considering our data, TNFα may increase NKCC1 expression and thus increase cellular susceptibility towards ammonia toxicity.

In conclusion, we show that TNFα is a critical component of hepatic ammonia metabolism and by the same token enhances NKCC1 expression and cerebral susceptibility to ammonia toxicity.

## Methods

### Mice

*Tnfa* ^*−/−*^, *Tnfrsf1b* ^*−/−*^ *mice* were purchased from Jackson Laboratories. *Tnfrsf1a* ^*−/−*^ were previously described^20^. *Glul x Alb-Cre* were previously described^9^. All mice were kept on a C57Bl/6 genetic background. Animals were kept under specific pathogen free conditions. For ammonia challenge animals were injected with 12 mmol/kg or 14 mmol/kg body weight ammonium acetate (Sigma-Aldrich, Deisenhofen, Germany) followed by monitoring of the animals over time, normalization of reflexes and spontaneous movements were considered as recovery from coma. 30 mg/kg of bumetanide or control vehicle was administrated intraperitoneally 5 minutes prior to injection of 14 mmol/kg body weight ammonium acetate. 200 ng of TNF (RandD systems, UK) was administrated intravenously, D-Gal was injected i.p. 15 minutes prior to TNFα. All animal experiments were reviewed and approved by the Landesamt für Natur, Umwelt und Verbraucherschutz NRW (Recklinghausen, Germany) and were performed in accordance to the German animal protection law.

### Blood and cerebrospinal fluid ammonia measurement

Ammonia was measured within 3 min after blood sampling from the right heart ventricle or retro-orbital venous sinus or cerebrospinal fluid from the brain using an Ammonia Checker II (Daiichi Kagaku Co. Ltd).

### Histology

Snap frozen hepatic tissue sections were stained with antibodies against GS (mouse, monoclonal; BD Biosciences) or rhesus family B glycoprotein (goat, polyclonal; Abcam) or ornithine aminotransferase (rabbit, polyclonal; Abcam) or GLT1 (rabbit polyclonal, Abcam) followed by staining with anti-rabbit or anti-mouse or anti-goat antibody and DAPI.

Histological analysis of brain tissue was performed as previously described^9^. Mice were killed by i.p. injection of pentobarbital and perfused with 20 mL of physiological saline, followed by perfusion with 250 mL of Zamboni’s fixative [4% (wt/vol) paraformaldehyde and 15% (vol/vol) saturated picric acid in 0.1 M PBS, pH 7.2, 4–6 °C]. Tissue, submerged in 20% (wt/vol) sucrose in PBS (24 h at 4 °C) until complete saturation, and finally frozen in precooled 2-methylbutane (Sigma–Aldrich) at −40 °C before being sliced into 50-μm-thick sections on a cryotome (Frigomobil; Leica). Sections were stained for 48 hours with anti-NKCC1 (rabbit, polyclonal; Cell signaling). All antibodies were diluted 1:500 in PBS containing 0.1% saponin (Sigma–Aldrich) and 5% BSA (GE healthcare). Primary antibodies were labeled for 48 hours with fluorochrome-coupled anti-mouse Cy3 or anti-rabbit FITC antibodies (1:500).

### RT-PCR

RNA purification and RT-PCR analyses were performed according to manufacturer’s instructions (Qiagen RNeasy Kit) and as previously described^55^. Gene expression of β-actin, Aqp4, Aqp8, Aqp9 was performed using FAM/VIC probes from Applied Biosystem. For urea cycle genes, cDNA was generated using a QuantiTect Reverse Transcription Kit (Qiagen). Real-time PCR was performed using the following primer sequences (Eurogene): argininosuccinate lyase (ASL) rev: 5′-CCA GTG GCT ACT TGG AGG ACA G-3′ and ASL for: 5′-CC TCA AGG GAC TTC CAA GCA C-3′, carbamoyl phosphate synthetase 1 (CPS-1) rev: 5′-GAT ACT GGA GAC AGC ACA CCA ATC-3′ and CPS-1 for: 5′-TAT GTT ACC TAC AAT GGC CAG GAG-3′, ornithine transcarbamylase (OTC) rev: 5′-TAA GGA TTT CCC TTG CAA TAA AGG-3′ and OTC for: 5′-CCA GAG TCA AGT ACA GCT GAA AGG-3′, succinate dehydrogenase complex subunit A (SDHA) rev: 5′-GTG GGA ATC CCA CCC ATG T-3′ and SDHA for: 5′-CTT CGC TGG TGT GGA TGT CA-3′. mRNA expression levels were normalized to mRNA expression levels of SDHA.

### GS Activity Assay

GS activity was measured as previously described^9^. Briefly tissue homogenates were incubated with reaction mixture containing 60 mmol/L L-Gln, 15 mmol/L hydroxylamine-HCl, 20 mmol/L Na-arsenite, 0.4 mmol/L adenosine diphosphate, 3 mmol/L MnCl_2_, and 60 mmol/L imidazol-HCl buffer (pH 6.8) at 37 °C. The reaction was stopped by adding 0.2 mol/L trichloroacetic acid, 0.67 mol/L HCl, and 0.37 mol/L FeCl_3_. The formed glutamyl hydroxamate was measured photometrically in the supernatant at 500 nm.

### Western blotting

Tissues were homogenized in lysis buffer containing (PBS, 1% w/v Triton-X (Sigma-Aldrich) and protease inhibitor, used according to the manufacturer protocol (Sigma-Aldrich). Blots were probed with anti-NKCC1 (1:1000, rabbit, polyclonal; Cell Signaling), HRP-conjugated anti-rabbit antibodies (1:5000, Cell Signaling), HRP-conjugated anti-β-ACTIN (1:2000, Cell Signaling). Densitometric analysis was performed with the Kodak Image Station 4400, using Kodak Molecular Imaging software.

### Statistical analysis

Data are expressed as mean ± S.E.M. Statistical significant differences between two different groups were analyzed using students t test. Statistical differences between several groups were tested using one-way ANOVA with additional Bonferroni or Dunnett’s post-tests. Statistically significant differences between groups in experiments involving more than one time point were calculated using two-way ANOVA (repeated measurements).

### Data Availability

No datasets were generated or analysed during the current study.

## Electronic supplementary material

Supplementary info
