# Supplementary material for: TNFα induced up-regulation of Na+,K+,2Cl− cotransporter NKCC1 in hepatic ammonia clearance and cerebral ammonia toxicity
Source: Sci Rep. 2017 Aug 11;7:7938. doi: 10.1038/s41598-017-07640-8 (PMC5554233; doi:10.1038/s41598-017-07640-8)
Supplement: Supplementary file 1 — Supplementary info [file 41598_2017_7640_MOESM1_ESM.pdf]

TNFα induced up-regulation of $Na^+,K^+,2Cl^-$ cotransporter NKCC1 in hepatic ammonia clearance and cerebral ammonia toxicity

Vitaly I. Pozdeev[1,2,*], Elisabeth Lang[1,*], Boris Görg[1], Hans J. Bidmon[3], Prashant Shinde[2], Gerald Kircheis[1], Diran Herebian[4], Klaus Pfeffer[5], Florian Lang[2,6], Dieter Häussinger[1,+], Karl S. Lang[7,+], and Philipp A. Lang[2,+,*]

Supplementary Figure 1

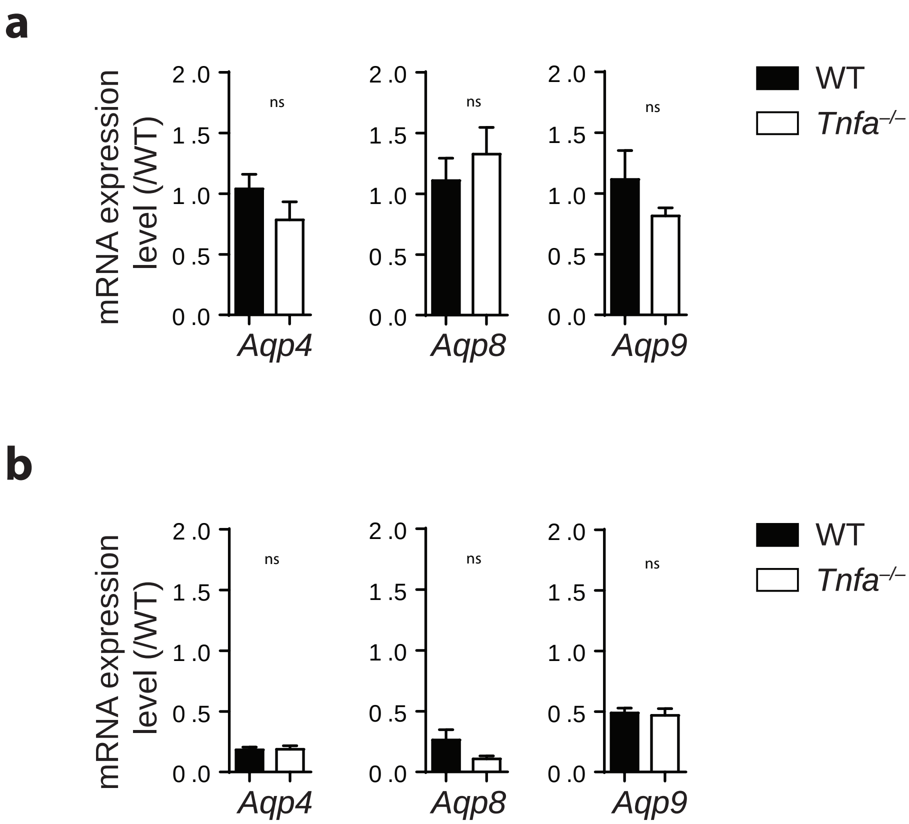

Suppl. Fig. 1: TNFα is dispensable for aquaporin expression. (A) RNA expression levels of Aqp4 (left panel), Aqp8 (middle panel), and Aqp9 (right panel) were measured in the cerebellum of WT and TNF deficient animals (n=6). (B) RNA expression levels of Aqp4 (left panel), Aqp8 (middle panel), and Aqp9 (right panel) were measured in the cortex of WT and TNF deficient animals (n=6-7).

Supplementary Figure 2

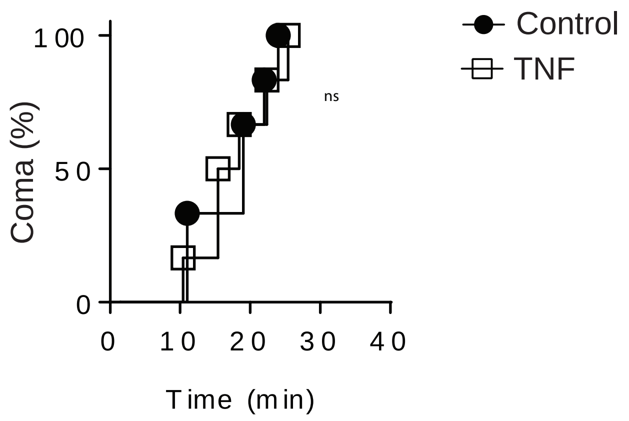

Suppl. Fig. 2: TNFα transiently promotes ammonia toxicity. C57Bl/6 animals were challenged intravenously with either 200ng TNF or vehicle. After 24 hours 12 mmol/kg ammonium acetate in PBS was injected intraperitoneally following measurement of the coma time (n=6).

Supplementary Figure 3

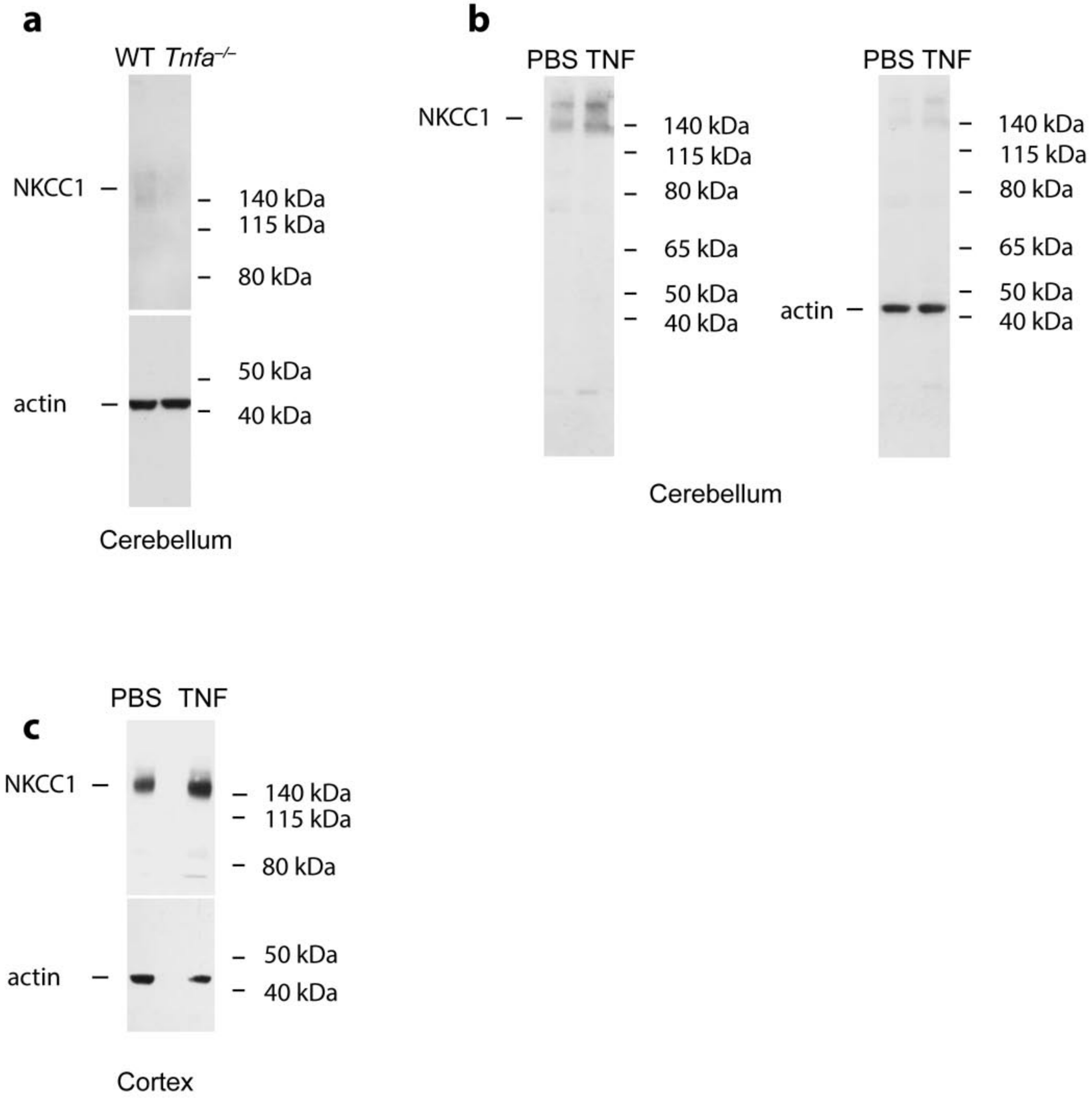

Suppl. Fig. 3: Uncropped Western blots shown in Figure 6. (A) Protein lysates harvested from the cerebellum of WT and *Tnfa*$^{-/-}$ mice were blotted and stained using anti-NKCC1 (upper panel) and anti-beta-actin (lower panel) antibodies (One representative of n=12 is shown) Cropped Western blots shown in Figure 6a.
(B,C) C57Bl/6 mice were treated with 200ng TNF. After 3h, protein lysates were prepared from (B) cerebellum or (C) cortex were blotted and stained with anti-NKCC1 and anti-beta-actin antibodies (left panels, one representative of n=8 is shown). Cropped Western blots shown in Figure 6c,e.
